# Comprehensive cardiac magnetic resonance T1, T2, and extracellular volume mapping to define Duchenne cardiomyopathy

**DOI:** 10.1186/s12968-023-00951-y

**Published:** 2023-07-31

**Authors:** Sudeep D. Sunthankar, Kristen George-Durrett, Kimberly Crum, James C. Slaughter, Jennifer Kasten, Frank J. Raucci, Larry W. Markham, Jonathan H. Soslow

**Affiliations:** 1 Thomas P. Graham Jr Division of Pediatric Cardiology, Department of Pediatrics, Vanderbilt University Medical Center, Monroe Carell Jr Children’s Hospital at Vanderbilt, 2220 Children’s Way, Suite 5230, TN 37232 Nashville, USA; 2 Department of Biostatistics, Vanderbilt University Medical Center, Nashville, TN USA; 3 Cincinnati Children’s Hospital Medical Center, Cincinnati, OH 45229 USA; 4 Division of Pediatric Cardiology, Department of Pediatrics, Children’s Hospital of Richmond at Virginia Commonwealth University Health System, Richmond, VA 23219 USA; 5 Division of Cardiology, Department of Pediatrics, Riley Hospital for Children at Indiana University Health, Indianapolis, IN 46202 USA

**Keywords:** Duchenne muscular dystrophy, Fibrosis, Edema, Parametric mapping, T1, T2, ECV

## Abstract

**Background:**

Cardiomyopathy is the leading cause of death in Duchenne muscular dystrophy (DMD). Cardiac magnetic resonance (CMR) parametric mapping sequences offer insights into disease pathophysiology. We propose a novel approach by leveraging T2 mapping in conjunction with T1 and extracellular volume (ECV) mapping to perform a virtual myocardial biopsy. While previous work has attempted to describe myocardial changes in DMD, our inclusion of T2 mapping enables comprehensive categorization of myocardial tissue characteristics of fibrosis, edema, and fat to better understand the pathological composition of the myocardium with disease progression.

**Methods:**

DMD patients (n = 49; median: 12 years-old) underwent CMR, including T1, T2, and ECV. Categories were defined as normal, isolated high T1 (normal ECV, high T1, normal T2), fibrosis (high ECV, normal or high T1, normal T2), edema (normal or high ECV, normal or high T1, high T2), fat (normal ECV, low T1, high T2) or fibrofatty (high ECV, low T1, high T2).

**Results:**

Median left ventricular ejection fraction (LVEF) was 59% with 27% having LVEF < 55%. Those with normal LVEF and no late gadolinium enhancement (37%) were younger in age (10.5 ± 2.6 vs. 15.0 ± 4.3 years-old, p < 0.001). Native T1 was elevated in at least one slice in 82% of patients. Those with high T2 at any slice (27%) were older (p = 0.005) and had lower LVEF (p = 0.005) compared with subjects with normal T2 (73%). The most common myocardial characterization was fibrosis (43%) followed by isolated high T1 (24%). Of the 13 with high T2, ten were categorized as edema, two as fibrofatty, and one as fat.

**Conclusion:**

CMR parametric mapping sequences offer insights into Duchenne cardiomyopathy pathophysiology, which should drive development of therapeutic interventions aimed at these targets. Myocardial fibrosis is common in DMD. Patients with elevated T2 were older and had lower LVEF. Though fat infiltration was present, the majority of subjects with elevated T2 met criteria for myocardial edema.

**Supplementary Information:**

The online version contains supplementary material available at 10.1186/s12968-023-00951-y.

## Introduction

Duchenne muscular dystrophy (DMD), an X-linked myopathy, affects approximately 1 in 5000 live male births [1–3]. The clinical phenotype of DMD can include muscular weakness, growth delay, cognitive impairment, respiratory failure, and cardiomyopathy. Cardiopulmonary disease is the most common cause of mortality in this population [4, 5]. With targeted ventilatory strategies improving life expectancy, prognostic and diagnostic cardiac testing has become increasingly critical [4–8].

The underlying pathogenesis of skeletal muscle disease is relatively well understood, with dystrophin loss leading to destabilization of the sarcolemma. The progression of disease has also been relatively well described, with recurrent muscle use leading to a cycle of cellular damage and repair that eventually leads to necrosis and fibrofatty replacement of skeletal muscle tissue. Presumably, the pathogenesis of myocardial progression is similar, but it is less well defined for a number of reasons: animal models do not completely mimic human disease and human cardiac autopsy and biopsy samples through the disease course have limited availability [9–11]. Prior work has postulated that late gadolinium enhancement (LGE) represents fibrosis, but the known fatty infiltration in skeletal muscle questions whether edema and fat play a role in progression of myocardial disease and specifically whether LGE by CMR is a combination of fat and fibrosis. Indeed, early reports from autopsy results from eight DMD patients consistently demonstrated fibrosis and fatty replacement of the myocardium with early fibrotic changes taking place at the epicardial-myocardium junction [11].

**Fig. 1:**
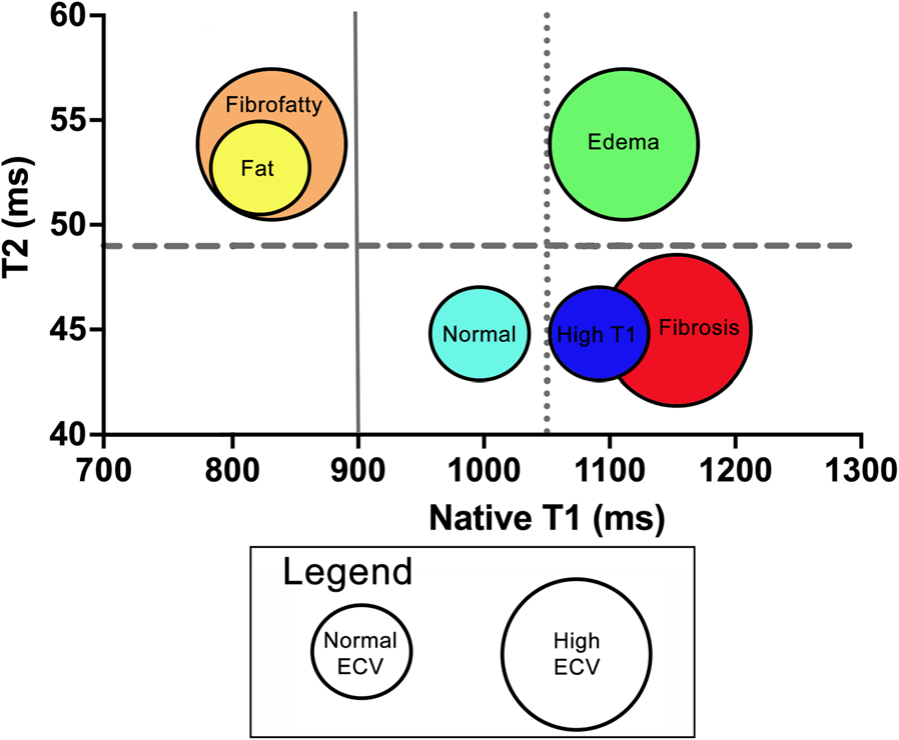
Myocardial changes defined by parametric mapping sequences. This figure demonstrates how T1, T2, and ECV can be utilized to define myocardial tissue composition. Quantification of T1, T2, and extracellular volume fraction (ECV) allow for characterization into normal, isolated high T1, fibrosis, fat, fibrofatty, and edema categories. Native T1 is on the X-axis, T2 is on the Y-axis, and ECV value is represented by the size of the circle. The cut-off for high and low native T1 is represented by the solid and dotted vertical lines (900 ms and 1050 ms, respectively). The dashed horizontal line indicates threshold for high versus normal T2 (49 ms). The cut-off for normal ECV is 28.5%. The overlap between the fibrosis and high T1 group in the figure represents the similar T1 and T2 times for these two categories, which are differentiated by ECV value. Similar methodology was applied for fat versus fibrofatty

Cardiac magnetic resonance (CMR) parametric mapping sequences, such as native T1, T2, and extracellular volume (ECV) mapping, allow for detailed tissue characterization and may allow for a more comprehensive assessment of DMD disease progression compared to echocardiography. Native T1 increases with fibrosis and edema and decreases with fat, while ECV increases with fibrosis and edema and remains unchanged with fat [12]. Previous work from our institution has shown higher mean native T1 and ECV in DMD patients compared to controls [13]. A recent publication demonstrated decreasing native T1 on serial CMR with increasing LGE, which the authors hypothesized was due to fatty replacement of myocardium [14]. However, this study was performed in a small population and did not include T2 mapping. T2 values increase with both fat and edema and the addition of T2 mapping gives additional, critical data to better understand myocardial disease progression. The combination of all 3 parameters in essence allows the performance of a non-invasive or “virtual” biopsy at various timepoints (Fig. 1).

Comprehensive parametric mapping, including T2 mapping, can help answer whether edema and fat play a role in the progression of DMD cardiovascular disease. These sequences can define the pathological progression of DMD cardiomyopathy, thus providing a roadmap for the future development of therapies that can arrest myocardial progression. Understanding the natural history of myocardial changes in this population may help direct timing and choice of pharmacologic intervention. More importantly, a better understanding of disease progression could help researchers identify novel, targeted therapies. Through this virtual biopsy, we aim to characterize the myocardial composition in DMD cardiomyopathy by evaluating the pathological changes that occur globally as well as in regions of interest with LGE and focal T2 elevation. We hypothesized that myocardial tissue characterization would demonstrate a progression similar to skeletal muscle, with evidence of edema, fibrosis, and fatty infiltration.

## Methods

### Patient selection

The Institutional Review Board approved this study. The majority of DMD subjects were enrolled prospectively (n = 45); additional DMD subjects who had signed consent for research CMR but were not enrolled in this specific prospective natural history study (n = 4) were also included. This natural history study was focused on collecting prospective clinical and CMR data for DMD boys to track longitudinal progression of disease. Enrollment was completed between November 2014 and November 2018. Consent was obtained for all participants; those under 18 years of age signed an age-appropriate assent form. DMD subjects able to undergo CMR without sedation or anesthesia with a clinical phenotype and confirmation with either genetic testing or muscle biopsy were included. Subjects were excluded from enrollment with a contraindication to CMR with contrast. Clinical data were collected from the electronic medical record.

### CMR acquisition and analysis

CMR was performed on a 1.5 Tesla Siemens Avanto (Siemens Healthcare Sector, Erlangen, Germany) or a 1.5 Tesla Siemens Avanto Fit. CMR protocol included functional imaging performed as previously described using balanced steady-state free precession imaging [15]. Intravenous gadolinium contrast was administered through a peripheral intravenous line (Gadobutrol 0.15 mmol/kg or gadopentate dimeglumine 0.2 mmol/kg). Late gadolinium enhancement (LGE) was performed using single shot inversion recovery (optimized inversion time to null myocardium) and phase sensitive inversion recovery (inversion time of 300 ms) imaging in the 4-chamber, 3-chamber, and 2-chamber planes as well as the short axis stack. Segmented inversion recovery (optimized inversion time to null myocardium) was also performed in the same slices as the parametric mapping. T2 and T1 mapping were performed in the same location at the base, mid-LV, and apex in the short axis plane, with T1 mapping repeated 15 min after contrast. All CMR post-processing was performed blinded to clinical data by an image analyst with all analyses verified by a cardiologist with > 10 years of experience (JHS). Ventricular volumes and function were calculated using Medis QMass (MedisSuite 2.1, Medis, Leiden, The Netherlands). T1, T2, and ECV maps were analyzed using QMaps (Medis). The T1, T2, and ECV for the entire slice (base, mid, and apex) were evaluated. Next, areas of LGE were localized on PSIR images and regions of interest (ROIs) were placed in these areas on the native T1, T2, and ECV maps. This was done to better characterize the pathologic changes associated with LGE. Finally, any focal areas of T2 elevation were identified on T2 maps and ROIs were placed on the T2 map as well as T1 and ECV maps. CMR acquisition and image post-processing is further described in the supplemental methods section.

**Fig. 2:**
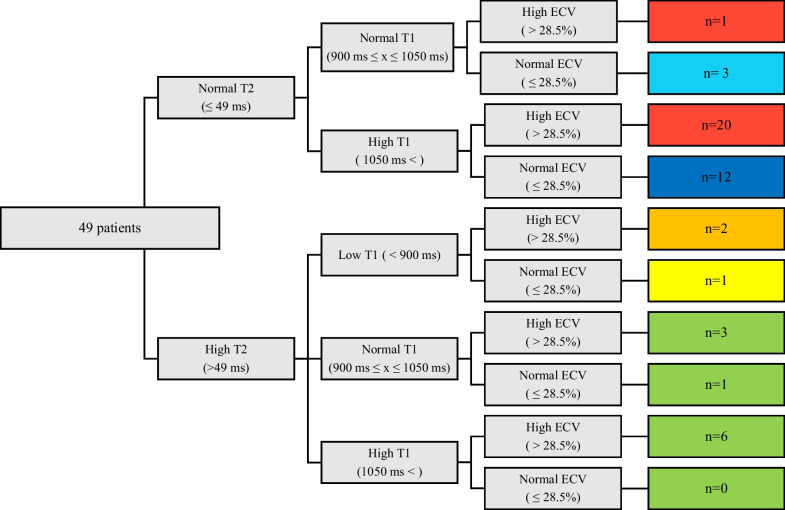
Myocardial characterization. Categorization performed with prespecified tissue characterization parameters. Initial dichotomization based on presence of high or normal T2 time with normal defined as ≤ 49 ms. Patients were then filtered by native T1 time (normal 900–1050 ms) and extracellular volume fraction (ECV; normal ≤ 28.5%). Color coding represents myocardial composition defined with light blue as normal, red as fibrosis, dark blue as isolated high T1, orange as fibrofatty, yellow as fat, and green as edema

Normal values for T1 and T2 were derived from 54 prospectively enrolled healthy controls of varying ages (range 7–56 years-old (y/o)) and sex (n = 29 male) as per the most recent parametric mapping consensus statement [12]. Normal values for ECV were obtained from the literature and a small cohort of local healthy controls [12, 16]. Patients were categorized by pre-specified T1, T2, and ECV parameters (Fig. 2) based on normal values. Normal parameters used for this study were T1 times between 900 and 1050 ms, T2 times < 49 ms, and ECV < 28.5%. These normal values were derived from normative data in our laboratory (native T1, T2, and ECV) as well as data reported in the literature (ECV only). For native T1, the normal range was defined as 2SD above and below the mean (the highest and lowest mean values from the base, mid, and apical slices were used in order to minimize false positives). For T2, the normal range was defined as 2SD above the highest mean. For ECV, a cut-off of 28.5% was chosen as this was 3SD above the mean and consistent with contemporary published reports from similar magnets. These values have been agreed upon by both pediatric and adult cardiologists at our institution to appropriately identify myocardial disease.

Individuals were first characterized by T2 values. Elevated T2 for any slice served as inclusion criteria for high T2 categorization. T1 values served as second tier characterization tool. Low T1 at any slice took precedent over T1 time at other slices categorizing the individual as low T1. If there was elevated T1 in any slice, without low T1 time at any slice, the individual was placed into the high T1 category. ECV percentage was dichotomized as normal or high based on a threshold of 28.5% [16, 17]. Figures 1 and 2 demonstrate how these three parametric mapping techniques were used to categorize individuals into normal, isolated high T1, fibrosis, fat, fibrofatty, or edema. A sub-analysis was performed with categorization for individual slices with longitudinal follow-up. Normative strain values were derived from our own institutional data and the literature [18]. Abnormal strain values were considered to be greater (less negative) than − 18%, which is our labs normal cut-off.

### Statistical analysis

**Fig. 3:**
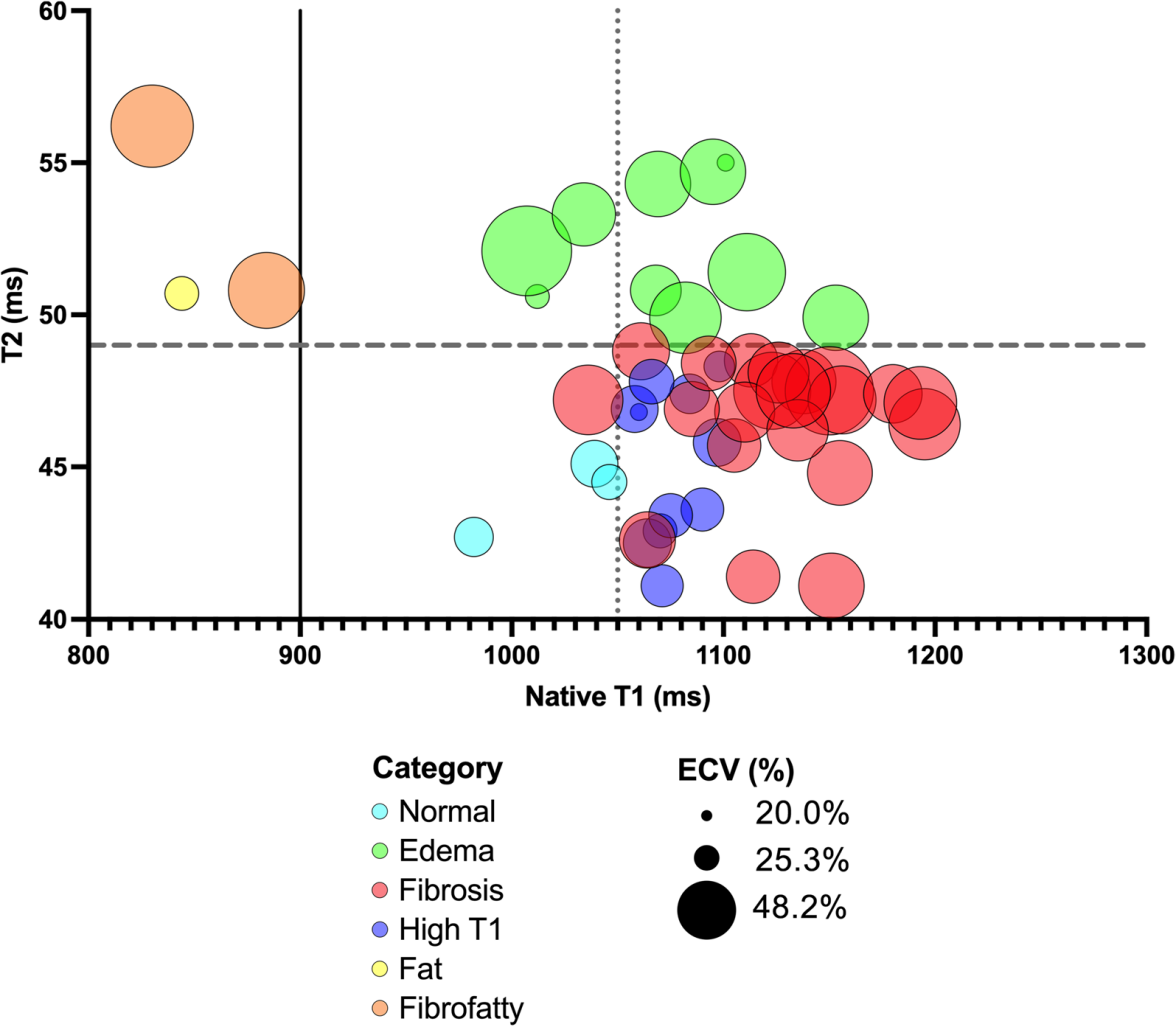
Scatterplot of tissue characterization parameters. Native T1, T2, and extracellular volume fraction (ECV) measurements for each patient. Native T1 is on the X-axis, T2 is on the Y-axis, and ECV value is represented by the size of the circle. The cut-off for high and low native T1 is represented by the solid and dotted vertical lines (900 ms and 1050 ms, respectively). The dashed horizontal line indicates threshold for high versus normal T2 (49 ms). Each patient categorization is represented by color as demonstrated in the legend and as explained in Fig. 1

Demographic variables were compared using Wilcoxon rank-sum test for continuous variables and Chi-square for categorical variables. Continuous variables compared between T2 groups were compared with Wilcoxon rank-sum test. Slice average tissue characterization parameters were compared to LGE and T2 ROIs within the same patient by Wilcoxon signed-rank test. Spearman’s rank correlation was used to estimate the correlation between continuous variables. Comparison of left ventricular ejection fraction (LVEF) and age between categories was completed with analysis of variance (ANOVA) followed by pairwise comparison of means with Tukey post-hoc testing. Statistical analysis was performed using Stata 16 (Stata Corporation, College Station, Texas, USA). Statistical significance was achieved with p < 0.05. Myocardial composition category plots (Figs. 1, 3) were created using Prism. All data were securely stored in a REDCap (Research Electronic Data Capture) database [19].

## Results

### Demographics

Forty-nine individuals with DMD met inclusion/exclusion criteria. The median age at first CMR was 12 years-old (interquartile range (IQR): 10.4–16.3). Full demographic characterization is available in Table 1.

**Table 1:** Patient characteristics

Characteristic	Value
Age	12.0 [10.4–16.3]
Height (cm)	145 [127–158]
Weight (kg)	47.8 [35.6–64.5]
BSA calculated (Haycock)	1.4 [1.2–1.7]
Race	
Caucasian African American Asian Other	43 (92%) 3 (6%) 2 (4%) 1 (2%)
Ethnicity	
Hispanic/Latino	6 (12%)
Medications	
Steroids ACEi Beta-blocker Aldactone ARB Aspirin Lasix	31 (63%) 26 (53%) 13 (27%) 6 (12%) 3 (6%) 3 (6%) 1 (2%)
LV ejection fraction (%)	59 [54–61]
LV cardiac index (L/min/m^2^)	3.5 [3.0–4.2]
Indexed LV end diastolic volume (ml/m^2^)	61 [55–73]
Indexed LV end systolic volume (ml/m^2^)	25 [22–32]
LV mass indexed (g/m^2^)	45 [41–52]
RV ejection fraction (%)	59 [55–62]
Late Gadolinium Enhancement Basal anterior Basal anteroseptal Basal inferoseptal Basal inferior Basal inferolateral Basal anterolateral Mid anterior Mid anteroseptal Mid inferoseptal Mid inferior Mid inferolateral Mid anterolateral Apical anterior Apical septal Apical inferior Apical lateral Apex	31 (63%) 11 (22%) 7 (14%) 7 (14%) 21 (43%) 28 (57%) 27 (55%) 9 (18%) 5 (10%) 9 (18%) 22 (45%) 30 (61%) 28 (57%) 10 (20%) 9 (18%) 11 (22%) 14 (29%) 7 (14%)

Patient characteristics and measurements at first cardiac MRI for 49 patients. Continuous variables presented as median [IQR] and categorical variables as count (frequency). *ACEi* Angiotensin converting enzyme inhibitor, *ARB* angiotensin receptor blocker, *BSA* body surface area, *LV* left ventricle, *RV* right ventricle

### CMR measurements and tissue characterization

Median LVEF was 59% with 13 patients having LVEF less than 55% at time of first CMR. Age was inversely related to LVEF (ρ = − 0.44, p = 0.002). The most common areas of LGE are shown in Table 1. Thirty-one DMD patients (63%) had at least one segment with LGE. Patients with LGE were older (median age 14.4 y/o vs.10.2 y/o; p < 0.001). As previously described, the basal and mid lateral walls were the most common locations for LGE, with the septal segments less often involved [20]. Using the full width half maximum technique, the median LGE in subjects with LGE was 28% IQR [15–35%].

High native T1 time was detected in at least one segment in 48 of 49 patients. Similar to the distribution of LGE, the highest areas of native T1 were the anterolateral and inferolateral segments of the basal and mid slices. Forty patients (82%) had at least one slice with elevated T1 with the base being the most common slice (base n = 33, mid n = 26, apex n = 21). Thirty-five patients (71%) had elevated T2 in at least one segment and 13 patients (27%) had elevated T2 in at least one slice at first CMR. The highest T2 values by slice and segment were seen at the apex (Table 2; Additional file 1: Table S1). While the base was the most common location for elevated T1, no individual had elevated T2 of the entire basal slice (focal elevations were detected at the base as described below). All 13 patients with elevated T2 had elevation in the apical slice, while four of these 13 patients had concomitant elevation at the mid slice. Average slice strain values were elevated (less negative or worse) at each slice compared to normal values, with a gradient from base to apex with apical strain more preserved (Table 2). There was no correlation between strain and parametric mapping values (T1, T2, and ECV). There was no significant difference between strain values between the different categories (Table 3). Use of steroids was not associated with a significant difference in strain values at the base, mid, or apex.

**Table 2:** Tissue characterization parameter measurements

	Native T1 (ms)	T2 (ms)	ECV (%)	Strain (%)
Global base	1072 [1037, 1100]	43.4 [41.8, 45.4]	29.0 [24.5, 34.0]	− 13.5 [− 15.9, − 12.1]
Global mid	1057 [1021, 1094]	44.4 [42.7, 46.0]	29.0 [24.6, 32.5]	− 15.5 [− 17.7, − 13.1]
Global apex	1047 [1000, 1099]	47.2 [45.1, 49.9]	29.8 [26.4, 34.0]	− 16.5 [− 18.6, − 14.0]

Characteristics at first MRI. Continuous variables presented as median [IQR]. ECV, extracellular volume fraction

**Table 3:** Characteristics by myocardial categorization

Characteristic	Normal (n = 3)	High T1 (n = 12)	Fibrosis (n = 21)	Edema (n = 10)	Fat (n = 1)	Fibrofatty (n = 2)
Age (years)	11.5 [8.5–14.3]	11.7 [9.8–14.7]	11.0 [9.6–15.0]	14.6 [12.0–21.4]	14.0	18.5 [17.8–19.1]
Weight (kg)	57.0 [33.2–66.5]	47.9 [40.2–70.1]	42.7 [30.5–52.3]	52.6 [38.1–82.7]	73.2	76.6 [64.5–88.6]
Steroid use at CMR1	3 (100%)	11 (92%)	12 (57%)	4 (40%)	0 (0%)	1 (50%)
LV Ejection Fraction (%)	66 [64–67]	60 [59–62]	59 [55–61]	55 [49–57]	68	36 [24–48]
LGE present	1 (33%)	5 (42%)	14 (67%)	9 (90%)	0 (0%)	2 (100%)
LGE (%)	9.77	12.5 [9.3–25.1]	30.2 [19.5–36.4]	28.2 [24.1–38.6]	–	44.7 [28.0–61.4]
Basal strain	− 17.2 [ − 19.1, − 15.0]	− 13.6 [− 15.7, − 12.6]	− 13.4 [− 15.8, − 12.4]	− 13.6 [− 16.1, − 10.6]	− 13.3	− 8.9 [− 12.1, − 5.6]
Mid strain (%)	− 18.6 [− 18.8, − 17.3]	− 16.7 [− 18.6, − 13.6]	− 14.6 [− 16.9, − 13.0]	− 14.7 [− 16.8, − 11.3]	− 15.5	− 8.5 [− 13.1, − 3.9]
Apical strain (%)	− 18.6 [− 19.7, − 18.1]	− 17.6 [18.9, − 14.6]	− 16.2 [− 18.0, − 13.5]	− 15.9 [− 19.2, − 12.3]	− 14.7	− 9.7 [− 15.2, − 4.2]

Characteristics based on proposed myocardial categorization at the time of first CMR. Continuous variables presented as median [IQR]; categorical variables presented as number (%). CMR1, first cardiac MRI; ECV, extracellular volume fraction; LGE, late gadolinium enhancement; LV, left ventricle

**Fig. 4:**
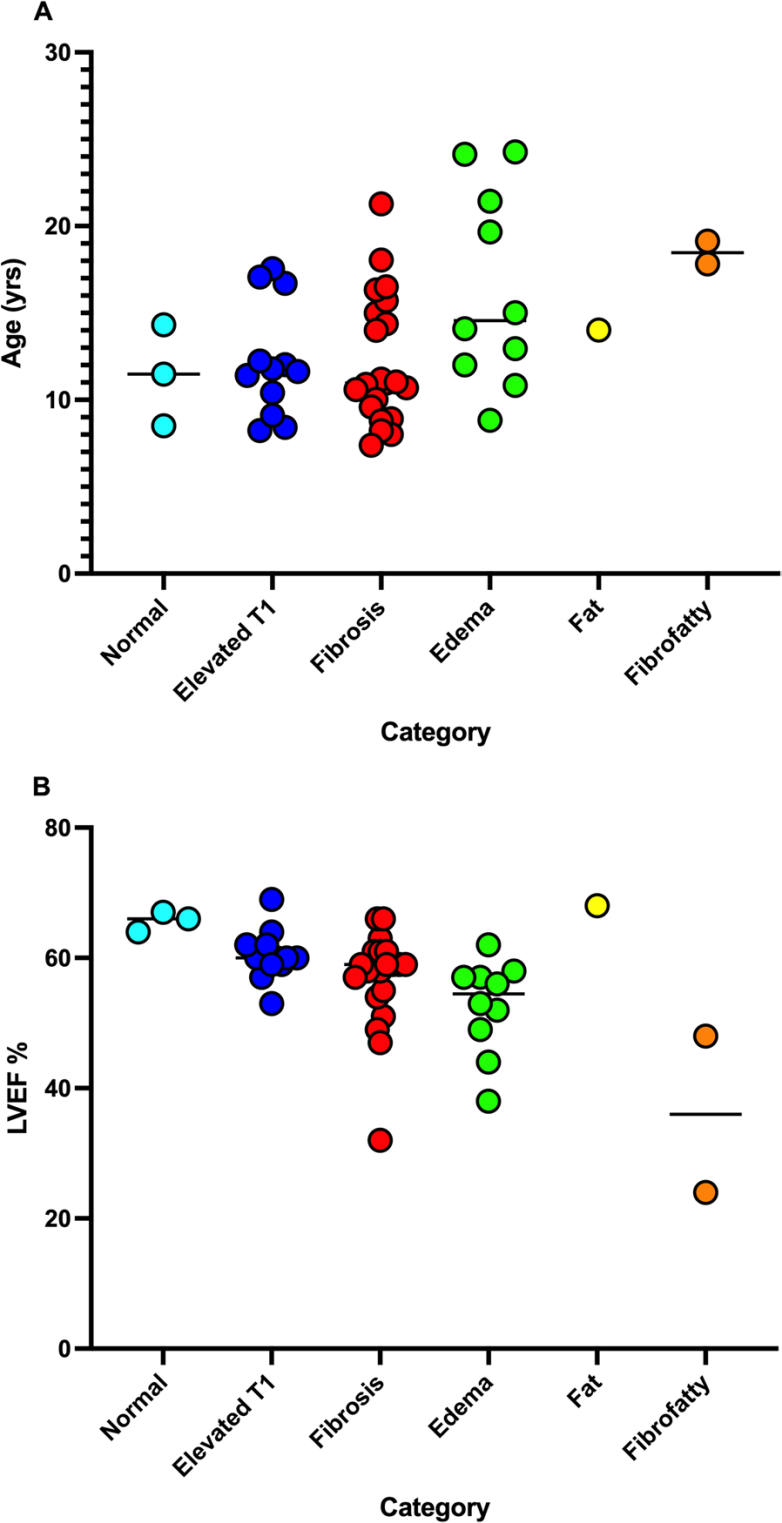
Scatterplot of age and LVEF by category. **A** Those with elevated T2 values (edema, fat, fibrofatty) were older (p = 0.005) than those with normal T2 values (isolated elevated T1, fibrosis) as determined by Wilcoxon rank-sum test. **B** Those with fibrofatty classification had significantly lower LVEF than all other groups. ANOVA was used to test for significance with Tukey post-hoc testing completed to detect intergroup difference

Figure 2 demonstrates the characterization performed utilizing T1, T2, and ECV. The most common characterization (Fig. 3) was fibrosis (n = 21/49, 43%) followed by isolated high T1 (n = 12/49, 24%). Of the 13 subjects with high T2, ten were categorized as edema, two as fibrofatty, and one as fat. Normal tissue characterization parameters were found in three patients. The difference in age between the categories did not reach statistical significance (p = 0.06; Table 3; Fig. 4A). There was a significant difference in LVEF between categories by ANOVA (p < 0.001; Table 3; Fig. 4B). Post-hoc testing demonstrated LVEF in the fibrofatty group was significantly lower when compared to each group (p < 0.05); however, no other inter-group LVEF difference was significant.

### Analysis by T2 characterization

Dichotomization was performed based on presence or absence of high T2 for at least one slice. Individuals with elevated T2 were older (15.0 y/o [12.9–19.7] vs. 11.3 y/o [9.4–14.7], p = 0.005), weighed more (65 kg [43–83] vs. 45 kg [33–57], p = 0.027), and had lower LVEF (53% [48–57] vs. 59% [58–62], p = 0.005) compared to those with normal T2 (Table 4). Those individuals taking steroids at the time of first CMR were less likely to have at least one slice with elevated T2 values (16% vs. 44%, p = 0.030). Native T1 times were lower in the mid and apex slices for the high-T2 group (999 ms [981–1069] vs. 1062 ms [1040–1094], p = 0.012; 1000 ms [905–1030] vs. 1063 ms [1026–1108], p = 0.009). ECV was elevated in the apex in individuals with elevated T2 (32% [31.0–35.0] vs. 29.0% [26.2–31.3], p = 0.032).

**Table 4:** Characteristics by T2 values

Characteristic	Normal T2 (n = 36)	High T2 (n = 13)	p-value
Age (years)	11.3 [9.4–14.7]	15.0 [12.9–19.7]	0.005
Weight (kg)	45 [33–57]	65 [43–83]	0.027
Height (cm)	142 [124–152]	160 [142–170]	0.016
LV ejection fraction (%)	59 [58–62]	53 [48–57]	0.005
LV Cardiac Index (L/min/m^2^)	3.6 [3.3–4.3]	3.1 [2.6–3.9]	0.035
Indexed LV diastolic volume (ml/m^2^)	60 [55–69]	73 [59–103]	0.068
Indexed LV systolic volume (ml/m^2^)	25 [22–30]	32 [25–50]	0.026
LV mass indexed (g/m^2^)	45 [41–48]	53 [45–65]	0.021
RV ejection fraction (%)	60 [55–64]	57 [55–60]	0.055
Indexed RV diastolic volume (ml/m^2^)	54 [49–66]	66 [62–76]	0.008
Indexed RV systolic volume (ml/m^2^)	23 [19–27]	29 [25–31]	0.002
Base native T1 (ms)	1073 [1050–1105]	1069 [1011–1088]	0.340
Mid native T1 (ms)	1062 [1040–1094]	999 [981–1069]	0.012
Apex native T1 (ms)	1063 [1026–1108]	1000 [905–1030]	0.009
ECV base (%)	27.5 [24.0–32.6]	32 [28.0–37.9]	0.060
ECV mid (%)	28.5 [24.4–31.5]	30 [27.5–36.0]	0.210
ECV apex (%)	29.0 [26.2–31.3]	32 [31.0–35.0]	0.032

Characteristics based on normal or high T2 values. Continuous variables presented as median [IQR]. Wilcoxon rank-sum test was used to detect a difference between groups for continuous variables. *ECV* extracellular volume fraction, *LV* left ventricle, *RV* right ventricle

### Longitudinal analysis

CMR parameters were compared between CMR-1 versus CMR-2 and CMR-3, respectively (Table 5). LVEF decreased over serial CMR. T2 times were significantly elevated on CMR-2 and CMR-3 compared to CMR-1 at each slice, though the difference between basal T2 for CMR 1 versus 3 did not reach statistical significance (p = 0.062). Native T1 and ECV times were not significantly different on follow-up CMR.

**Table 5:** Characteristics compared over serial CMR

	CMR 1 vs 2	CMR 1 vs 3
Left ventricular ejection fraction (%)	2.6 ± 0.5 < 0.001	4.5 ± 0.8 < 0.001
Indexed LV end diastolic volume (ml/m^2^)	− 0.98 ± 1.4 0.484	− 3.1 ± 1.8 0.093
Indexed LV systolic volume (ml/m^2^)	− 2.3 ± 0.9 0.014	− 4.3 ± 1.0 < 0.001
LV mass indexed (g/m^2^)	3.3 ± 1.0 0.003	6.7 ± 1.3 < 0.001
Right ventricular ejection fraction (%)	1.3 ± 0.9 0.163	1.7 ± 0.9 0.079
Basal strain (%)	− 0.82 ± 0.5 0.095	− 0.82 ± 0.5 0.115
Mid strain (%)	− 0.47 ± 0.5 0.371	− 0.81 ± 0.5 0.115
Apical strain (%)	− 0.41 ± 0.6 0.463	− 0.25 ± 0.6 0.700
Global base native T1 (ms)	2 ± 9 0.780	10 ± 10 0.325
Global mid native T1 (ms)	20 ± 12 0.107	17 ± 12 0.164
Global apex native T1 (ms)	12 ± 15 0.412	46 ± 22 0.048
Global base T2 (ms)	− 1.9 ± 0.5 < 0.001	− 1.0 ± 0.5 0.062
Global mid T2 (ms)	− 1.6 ± 0.4 < 0.001	− 1.5 ± 0.7 0.029
Global apex T2 (ms)	− 1.7 ± 0.6 0.011	− 3.2 ± 1.4 0.025
Global base ECV (%)	− 0.8 ± 0.6 0.198	− 0.7 ± 0.7 0.338
Global mid ECV (%)	− 0.2 ± 0.8 0.728	0.0 ± 0.9 0.997
Global apex ECV (%)	0.2 ± 0.8 0.782	0.5 ± 1.0 0.613

Mean difference and standard error between serial CMR and tissue characterization parameter measurements. The first row in the cell is the mean difference with positive values indicating a higher value measured with CMR1 than the compared study. The second row is the p-value. *ECV* extracellular volume fraction, *LV* left ventricle, *RV* right ventricle

**Fig. 5:**
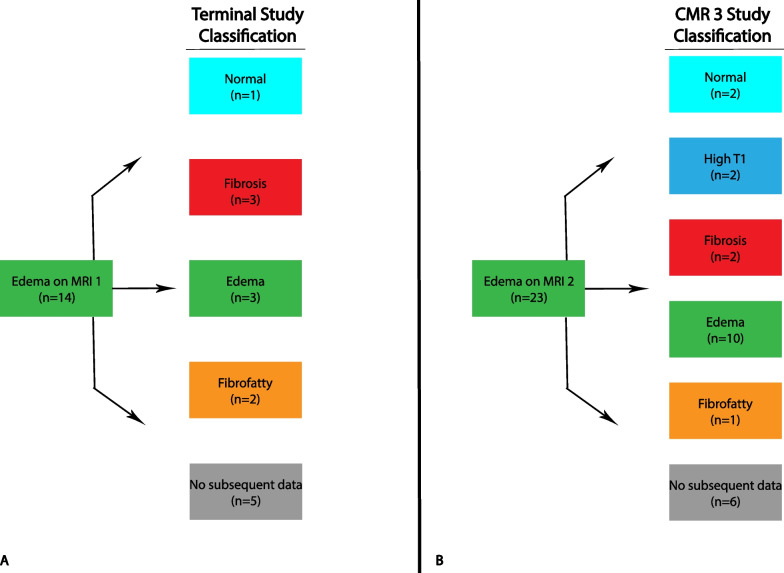
Progression of slice edema on serial CMR. **A **Subjects with edema on CMR-1 for any slice (base, mid, or apex, represented as elevated T2 and normal or high T1 for the entire slice) are demonstrated with their categorization designation on terminal CMR available for review. **B** Includes subjects with slice edema detected on CMR-2 and without edema on CMR-1, as well as their cardiovascular progression as illustrated by their categorization on CMR-3. There is no overlap in slices included between (**A**) and (**B**). Those without follow-up CMR are designated as “no subsequent data”

Sub-analysis detailing individual slice progression was also performed with attention to slices demonstrating edema. There were 10 patients with 14 slices of edema (n = 10 at apex, n = 4 at mid) on initial CMR (Fig. 5A). Of these, only one slice had a normal classification on terminal CMR; however, this slice had a top normal T2 time of 48.7 ms. The second CMR had 19 patients with 23 slices of edema (n = 4 at base, n = 3 at mid, and n = 16 at apex) (Fig. 5B). Comparison with the same slice locations on the first CMR revealed that all these slices represented new edema. Among these 23 slices, only two were classified as normal on the third CMR with one of these two patients having a top normal T1 time of 1050 ms. Amongst the ten patients with edema on CMR-1, six were not on steroids at initial CMR. Only one patient from this subgroup had steroids added in the interval between first and second CMR. This patient had edema on CMR-2 and had elevated T1 on CMR-3. One patient without prior steroid use and with fat infiltration on CMR-1 was started on steroids before CMR-2 and had progression to edema and then fibrosis on CMR-2 and CMR-3, respectively.

### ROI analysis

Areas of LGE were localized on PSIR images and ROIs were placed in these areas on the native T1, T2, and ECV maps (basal n = 29; mid n = 30). LGE ROIs demonstrated higher native T1, T2, and ECV at base and mid, though the difference in mid T1 did not reach statistical significance (Additional file 1: Table S2).

**Fig. 6:**
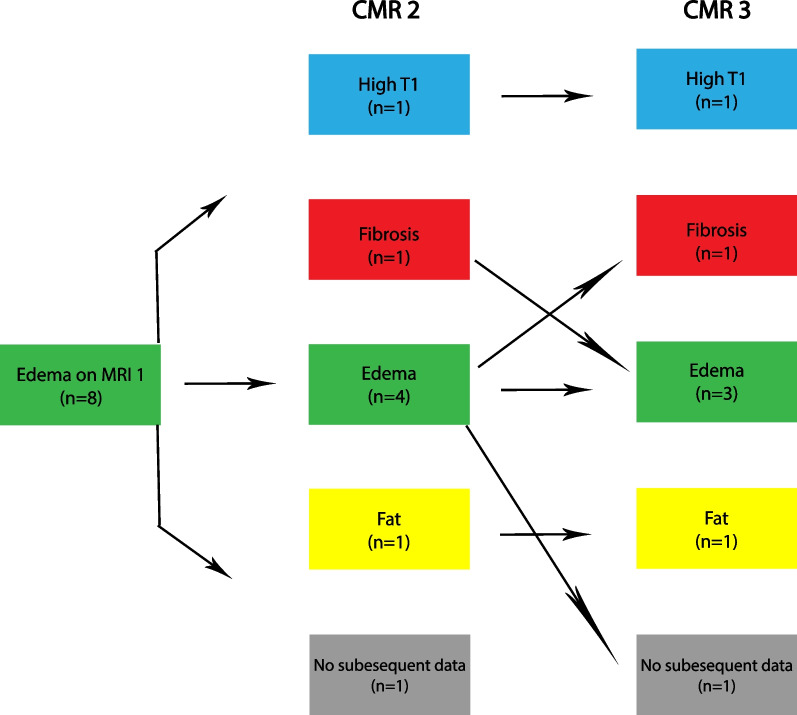
Progression of edema for base T2 ROI. Eight individuals had edema detected on CMR-1 in the base T2 ROI. Progression of these eight patients on CMR-2 and 3 is demonstrated. Those without follow-up CMR are designated as “no subsequent data”

**Fig. 7:**
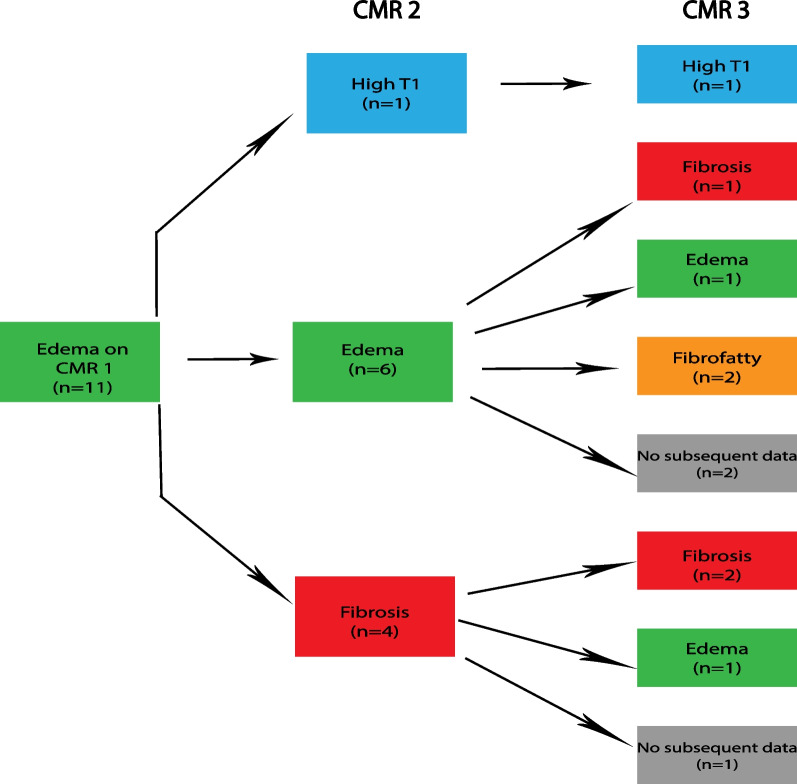
Progression of Edema for Mid T2 ROI. Eleven individuals had edema detected on CMR-1 in the mid T2 ROI. Progression of these 11 patients on CMR-2 and 3 is demonstrated. Those without follow-up CMR are designated as “no subsequent data”

Areas of focal T2 elevation were identified on T2 maps and ROIs were placed in these areas on T2, native T1, and ECV maps. T2 ROIs were identified in 16 patients (8 with T2 elevation at basal slice and 13 at mid slice). These ROIs demonstrated elevated ECV and T1, though basal T1 did not reach statistical significance (Additional file 1: Table S3). These ROIs were classified as edema in 94% (n = 15/16) at the initial CMR. Follow up CMR evaluation in the same location demonstrated abnormal parametric mapping in all cases, with persistence of edema or development of fibrosis being the most common findings (Figs. 6, 7).

## Discussion

**Fig. 8:**
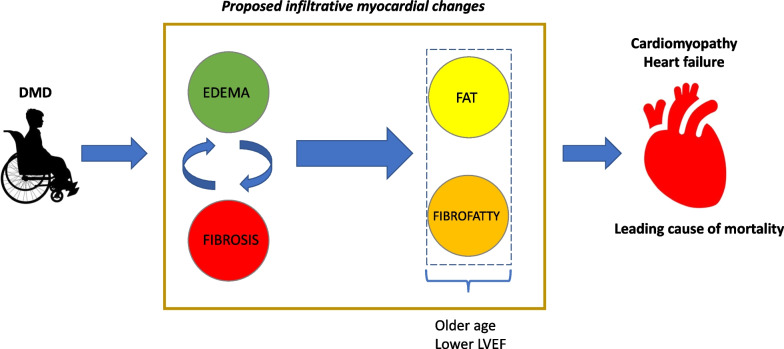
Postulated myocardial infiltrative changes. Edema and fibrosis were the most common myocardial changes detected in this study. We postulate that there is a cyclical interchange between edema and fibrosis with fat and fibrofatty replacement as the terminal myocardial composition

This is the first study of which we are aware to use advanced parametric mapping techniques, including newer T2 mapping sequences, to classify DMD subjects and perform a “virtual myocardial biopsy” to better understand characteristics of myocardial disease and progression. The primary findings of our study are: (1) fibrosis is the most common DMD myocardial composition change, and normal myocardial tissue characterization is rare in DMD; (2) elevated T2 times occur in older patients with decreased LVEF; and (3) most areas of LGE are comprised predominately of fibrosis. The majority of patients with elevated T2 at the slice level had elevated T1 and ECV in the same location. Taken together, these data suggest that edema is a common pathway in the development of myocardial abnormalities and that fatty infiltration, while present, likely occurs in older patients with more significant myocardial disease (Fig. 8).

Studies have shown increased T2 relaxation time in thigh muscles of DMD patients in comparison to controls [21]. With the belief that skeletal muscle pathogenesis parallels that of the cardiac muscle, we suspected T2-mapping to play an important role in understanding progression of myocardial disease. Previous studies utilizing a black blood dual spin method for T2 acquisition demonstrated higher T2 times in older patients with decreased LVEF corroborating our findings [22]. While findings are similar, our study utilizes a more contemporary T2 mapping sequence to analyze the myocardium. Interestingly, there was no elevation in T2 at the basal slice in any patient and the apical slice was the most commonly affected slice. LGE data suggest that the basal and mid slices are the first to develop LGE with a progression from base to apex. We hypothesize that T2 is more commonly elevated at the apex as these regions are more commonly undergoing the cycle of inflammation/edema in older boys undergoing CMR, while the basal and mid slices have had a significant portion of myocardium already replaced with fibrosis. In support of this, there remain ROIs of elevated T2 at the base and mid slices, but these focal areas of edema are unable to increase the average T2 into the abnormal range. Longitudinal follow-up for slices with edema, in this cohort, demonstrated that once edema is present, return to normal myocardial composition is rare. This is clinically relevant as the presence of myocardial inflammation leading to longitudinal decreased ventricular function has been previously described in the DMD population; we hypothesize that the myocarditis/dystrophinitis reported in these publications represents an extreme myocardial inflammatory response due in part to natural progression of DMD myocardial disease [23, 24]. T2 ROIs showed absence of normal regions following the presence of edema on initial CMR. Persistence of edema or progression to fibrosis were the most common subsequent findings with few demonstrating fibrofatty infiltration. These findings suggest that once edema is present, there is cyclical occurrence of edema and fibrosis until late fatty deposition is detected (Fig. 8). Although diffuse fibrosis can improve, replacement fibrosis is unlikely to resolve. Unfortunately, these imaging techniques are unable to distinguish between edema alone and edema with underlying fibrosis. Late follow-up CMR and a larger sample size of fat or fibrofatty deposition would be beneficial in corroborating this supposition. Finally, we hypothesize that fatty replacement is more likely to occur first in basal slices, as the ROIs of elevated T2 at the base had no difference in native T1 (and in fact a trend towards lower T1) suggestive of a mix of both edema and fat.

Global T1 is lower in patients with elevated T2; however, in ROIs of T2 elevation at the mid slice, the T1 tended to be elevated. We hypothesize that fatty infiltration is a more diffuse process, while edema is more focal. We also suspect, based on the pathogenesis of disease, that edema is a transient finding during early myocardial inflammation and/or ischemia and thus more difficult to capture prior to fibrotic changes. Emerging pre-clinical data suggest that edema may be part of earlier pathogenesis, while fatty infiltration is a late finding [25]. With only three patients classified as having fatty or fibrofatty infiltrate, larger cohorts and more longitudinal data will be necessary to substantiate this theory.

Elevated native T1 and ECV have been demonstrated in patients with DMD previously [13, 26, 27]. In this cohort, T1 times were most commonly increased in the basal and mid slices with highest values in the inferolateral segment of each of these slices. This segment has been demonstrated to be affected in non-ischemic myocarditis as well as in our previous studies of DMD [13, 28]. While the reason early myocardial abnormalities localize to this segment is unclear, focusing on this region may allow for earlier detection of disease.

Although 98% of this cohort demonstrated elevated T1 in at least one segment, it is likely that many of these abnormalities are spurious. Indeed, previous studies from our institution have shown that even healthy controls may have segmental T1 abnormalities [29]. It is possible that this could represent transient inflammation or edema, even in healthy controls. There have been reports of myocarditis superimposed on DMD cardiomyopathy [30, 31]. This may be more pertinent in DMD as they do not have normal cardiomyocyte reserve that otherwise normal patients have. While segmental abnormalities could be due to chance, 82% of patients demonstrating at least one slice with elevated T1 is more likely due to early myocardial disease. Although the prognostic significance of elevated T1 is unclear, the elevation of T1 prior to development of LGE has been described in prior cohorts [13, 20, 26].

Strain data in this manuscript suggest a similar base to apex gradient as seen with LGE. While strain values did not correlate with parametric mapping values in this cohort, there were abnormal strain values at all three slices. Strain values remained abnormal over serial CMR; however, there was no significant change in strain values over serial CMR in this cohort. While the focus of this study was on parametric mapping values to categorize myocardial changes, it is clear that longitudinal strain values should be followed with patients demonstrating abnormal strain with baseline CMR. The authors believe utilizing strain to perform 3D myocardial mapping could allow for better understanding of segmental remodeling and is an area of future exploration.

### Limitations

Parametric mapping changes are not seen uniformly throughout the myocardium; therefore, assignment to a particular characterization group may only partially describe the pathology present. The truth is likely that there is patchy progression and that multiple different pathological changes are present in some patients. Unfortunately, the parametric mapping methods reported here are unable to distinguish these more complex tissue patterns, which would be apparent in an actual myocardial biopsy. The authors acknowledge that current parametric mapping techniques cannot fully replace information obtained from an actual biopsy; however, we believe that cardiac MRI may serve as a useful non-invasive method to help understand cardiomyopathy progression in DMD that has, up to this point, been difficult to assess due to limited tissue samples. In addition, these methods are ideal for tracking longitudinal myocardial changes.

Due to technical limitations, we were unable to complete fat/water separation imaging to provide additional evidence to distinguish between edema and fat infiltration, but we feel that the combination of T1, T2, and ECV provides an adequate, quantitative method for tissue characterization. Many DMD patients become uncomfortable in the scanner due to contractures, scoliosis, or claustrophobia. Those that were unable to tolerate CMR were not enrolled and this could lead to some bias in the study, though these factors are usually unrelated to cardiac disease. In addition, given the concern for discomfort, our DMD protocols are designed to limit required scan time. This comes at the expense of additional sequences. Therefore, long axis imaging (2-, 3-, and 4-chamber) and more comprehensive short axis coverage were not performed in an effort to limit total scan time for our patients. More comprehensive coverage could not be performed without removing other sequences of interest (tagged images, comprehensive LGE, etc.). Therefore, there were areas of LGE without a map. However, given that LGE is usually present in contiguous slices, the “missed” areas were felt to be of minimal consequence.

Of note, partial volume averaging is a possible confounder for parametric mapping, particularly at the apical slices. We were careful to not contour slices felt to be “too apical” and to contour only the mid-myocardium. Blood pool in the ventricular cavity has high native T1 signal; therefore, if partial volume averaging were occurring, we would have expected artificially elevated T1 times at the level of the apex. The T1 values at the apex were lower in this study, suggesting that partial volume averaging was successfully avoided at the apical slices. Due to the inherent challenges with mapping of the apical slice, there were five apical slices that were unable to be analyzed for native T1 and six apical slices that were unable to be analyzed for ECV. All apical slices were able to be analyzed for T2.

## Conclusion

While LGE is unable to distinguish between fibrosis, fat, and edema, parametric mapping, including T2-mapping, may allow for the differentiation of these tissue characteristics. In this cohort of DMD subjects, fibrosis was the most commonly observed myocardial change. Patients with elevated T2 were older and had decreased LVEF, and the areas of LGE predominately represented fibrosis. Longitudinal data suggest that development of edema portends persistence of myocardial pathology with subsequent normalization of tissue parameters a rare finding. Measurement of T1, ECV, and T2 may provide a virtual biopsy to define the pathological composition of DMD cardiomyopathy with the eventual goal to develop targeted therapies to mitigate these changes.

## Supplementary Information

**Additional file 1: Table S1**: Segmental T1 and T2 Measurements. **Table S2**: Tissue Characterization Parameters in LGE ROIs. **Table S3**: Tissue Characterization Parameters in T2 ROIs.

## Data Availability

The datasets used and/or analyzed during the current study are available from the corresponding author on reasonable request.

## Abbreviations

ACEi: Angiotensin converting enzyme inhibitor
ANOVA: Analysis of variance
ARB: Angiotensin receptor blocker
BSA: Body surface area
CMR: Cardiac magnetic resonance imaging
DMD: Duchenne Muscular Dystrophy
ECG: Electrocardiogram
ECV: Extracellular volume
IQR: Interquartile range
LGE: Late gadolinium enhancement
LV: Left ventricle
LVEF: Left ventricular ejection fraction
MOLLI: Modified Look-Locker inversion recovery
REDCap: Research Electronic Data Capture
ROI: Regions of interest
