# Supplementary material for: Comprehensive cardiac magnetic resonance T1, T2, and extracellular volume mapping to define Duchenne cardiomyopathy
Source: J Cardiovasc Magn Reson. 2023 Jul 31;25:44. doi: 10.1186/s12968-023-00951-y (PMC10388519; doi:10.1186/s12968-023-00951-y)
Supplement: Supplementary file 1 — Additional file 1: Table S1: Segmental T1 and T2 Measurements. Table S2: Tissue Characterization Parameters in LGE ROIs. Table S3: Tissue Characterization Parameters in T2 ROIs. [file 12968_2023_951_MOESM1_ESM.docx]

**Additional file 1: Appendix**

**Additional file 1: Methods**

*CMR Acquisition*

CMR was performed on a 1.5 Tesla Siemens Avanto (Siemens Healthcare Sector, Erlangen, Germany) with an 8-channel cardiac coil or a 1.5 Tesla Siemens Avanto Fit (Siemens) with a 32 channel coil. CMR protocol included functional imaging performed as previously described using balanced steady-state free precession imaging.(15) Intravenous gadolinium contrast (gadopentate dimeglumine, Magnevist®, Bayer Healthcare Pharmaceuticals, Wayne, New Jersey, USA at a dose of 0.2mmol/kg or gadobutrol, Gadavist®, Bayer Healthcare Pharmaceuticals, Wayne, New Jersey, USA at a dose of 0.15mmol/kg) was administered through a peripheral intravenous line. Late gadolinium enhancement (LGE) was performed using single shot inversion recovery (optimized inversion time to null myocardium) and phase sensitive inversion recovery (inversion time of 300ms) imaging in the 4-chamber, 3-chamber, and 2-chamber planes as well as the short axis stack. Segmented inversion recovery (optimized inversion time to null myocardium) was also performed in the same slices as the parametric mapping.

T2 mapping was performed at the base, mid-ventricular level, and apex in the short axis plane at the same slice location as the cine and LGE imaging. T2 mapping was performed as a breath-held, electrocardiogram (ECG)-triggered, bSSFP sequence with motion correction. Typical imaging parameters were as follows: Adiabatic T2 preparation with 35-degree flip angle, field of view 340 x 272 mm^2^, matrix size 192 x 144, slice thickness 8mm, voxel size 1.8 x 1.9 x 8.0 mm^3^, TR/TE 2.5ms/1.1ms, parallel imaging factor of 2.

Breath-held modified Look-Locker inversion recovery (MOLLI) sequences were performed prior to and 15 minutes after contrast administration at the base, mid-ventricular level, and apex in the short axis plane at the same slice location as the T2 mapping.(32, 33) MOLLI sequences were motion-corrected, ECG-triggered images obtained in diastole with typical imaging parameters: non-selective inversion with a 35 degree flip angle, single shot SSFP imaging, initial inversion time of 120ms with 80ms increments, field of view 340 x 272 mm^2^, matrix size 256 x 144, slice thickness 8mm, voxel size 1.3 x 1.9 x 8.0 mm^3^, TR/TE 2.6ms/1.1ms, parallel imaging factor of 2. The matrix size was decreased to 192 x 128 for heart rates >90 (approximate voxel size 1.8 x 2.1 x 8 mm^3^). The pre-contrast MOLLI acquired 5 images after the first inversion with a 3 second pause (approximated by using a 3-beat pause for subjects with a heart rate of 60, a 4-beat pause for subjects with a heart rate of 80, and so on) followed by 3 images after the second inversion, or 5(3s)3. The post-contrast protocol was acquired at a 4(1)3(1)2.(34) Motion correction was performed and a T1 map was generated on the scanner.(35) A goodness of fit map was also performed at the time of the scan to evaluate data quality. Any image felt to be inadequate due to poor breath holds or poor motion correction was repeated at the time of the scan.

**CMR Post-Processing**

All CMR post-processing was performed blinded to clinical data by an image analyst with all analyses verified by a cardiologist with 10 years of experience (JHS). Ventricular volumes and function were calculated using Medis QMass (MedisSuite 2.1, Medis, Leiden, The Netherlands). The presence or absence of LGE, as well as location using the standard 17-segment model,(36) was qualitatively assessed. Percent LGE was calculated using the full width half maximum technique using QMass on the phase sensitive inversion recovery images as per our labs standard protocol.

T1 maps, obtained prior to and after contrast administration as described by Messroghli et al,(32) were used along with the subject’s hematocrit (obtained prior to CMR) to calculate an extracellular volume (ECV) map using manual registration in QMap from Medis. The ECV was calculated as:

$$ECV= \frac{\left( \frac{1}{{myocardialT1}_{\mathrm{post}}} \right)-\left( \frac{1}{{myocardialT1}_{\mathrm{pre}}} \right)}{\left( \frac{1}{{bloodpoolT1}_{\mathrm{post}}} \right)-\left( \frac{1}{{bloodpoolT1}_{\mathrm{pre}}} \right)}\left( 1-Hematocrit \right)$$

In cases where registration could not be adequately performed, native T1 and post-contrast T1 were traced separately and ECV calculated manually. Regions of interest (ROIs) were manually drawn on T1 and ECV maps within the LV mesocardium in the standard 16 segments, carefully avoiding partial volume averaging with blood-pool or epicardial fat. Areas of LGE were included as these areas were felt to be the most focal areas in a continuum of diffuse ECM expansion.(33) Imaging artifact was not contoured. Segments were not included in the analysis if the bounds of the myocardium could not be distinguished from surrounding tissue and blood pool or if image registration was inadequate in those segments. All regions of interest were traced by the image analyst.

In addition to the segmental ROIs and ROIs of the basal, mid-ventricular, and apical slices, the segmented LGE images were carefully examined to evaluate for presence of LGE. Up to 3 ROIs at the basal and mid slices were then placed in identical locations on the native T1, ECV, and T2 maps to perform tissue characterization in these areas of LGE. Finally, T2 maps were carefully examined for any areas of obvious elevation, denoting edema or fibrosis. The phase images were inspected to ensure that these areas were not a result of artifact. A ROI was placed in these areas and in an identical location on the native T1 and ECV maps. Up to 3 ROIs for T2 were identified per basal and mid slice. The average T1, T2, and ECV was calculated for each slice for each patient for LGE and T2 ROIs.

**Additional file 1 Tables**

**Table S1: Segmental T1 and T2 Measurements**

| Segment | Native T1 | T2 |
| --- | --- | --- |
| Basal anterior | 1051 [1017-1081] | 43.8 [41.5-45.9] |
| Basal anteroseptal | 1062 [1043-1098] | 43.4 [41.2-45.2] |
| Basal inferoseptal | 1044 [1024-1071] | 43.2 [40.9-44.6] |
| Basal inferior | 1063 [1026-1113] | 43.3 [41.6-45.0] |
| Basal inferolateral | 1084 [1051-1178] | 44.1 [40.5-45.9] |
| Basal anterolateral | 1069 [1047-1112] | 43.1 [41.1-45.1] |
| Mid anterior | 1032 [999-1076] | 44.3 [42.9-47.6] |
| Mid anteroseptal | 1052 [1011-1086] | 43.5 [41.4-46.2] |
| Mid inferoseptal | 1059 [1017-1079] | 43.7 [42.2-44.7] |
| Mid inferior | 1052 [1024-1101] | 43.7 [42.4-46.7] |
| Mid inferolateral | 1088 [1028-1155] | 43.7 [40.8-47.5] |
| Mid anterolateral | 1040 [986-1089] | 43.9 [42.5-47.4] |
| Apical anterior | 1022 [970-1081] | 48.4 [45.1-51.3] |
| Apical anteroseptal | 1067 [1033-1099] | 48.6 [45.4-50.8] |
| Apical inferior | 1029 [979-1087] | 45.7 [43.8-48.2] |
| Apical inferolateral | 1065 [964-1157] | 46.3 [43.5-50.2] |

Table S1 Caption: Native T1 and T2 times for each cardiac segment presented as median [IQR] for the first cardiac MR (CMR-1).

**Table S2: Tissue Characterization Parameters in LGE ROIs**

| Location / Parameter | Slice Average | LGE ROI | p-value |
| --- | --- | --- | --- |
| Base Native T1 (ms) | 1072[1037-1100] | 1109[1065-1151] | 0.014 |
| Base T2 (ms) | 43.4[41.8-45.4] | 45.2[43.4-47.8] | 0.002 |
| Base ECV (%) | 29.0[24.5-34.0] | 41.7[36.8-47.1] | < 0.001 |
| Mid Native T1 (ms) | 1057[1021-1094] | 1076[1038-1126] | 0.054 |
| Mid T2 (ms) | 44.4[42.7-46.0] | 47.3[43.1-49.8] | 0.018 |
| Mid ECV (%) | 29.0[24.6-32.5] | 38.5[35.3-43.1] | < 0.001 |

Table S2 Caption: Tissue characterization parameters in LGE ROIs. Continuous variables presented as median [IQR]. Wilcoxon signed-rank test was used to detect a difference between groups for continuous variables. ECV, extracellular volume fraction; LGE, late gadolinium enhancement; ROI, region of interest.

**Table S3: Tissue Characterization Parameters in T2 ROIs**

| Location / Parameter | Slice Average | T2 ROI | p-value |
| --- | --- | --- | --- |
| Base Native T1 (ms) | 1072[1037-1100] | 1049[976-1159] | 0.600 |
| Base T2 (ms) | 43.4[41.8-45.4] | 53.5[52.7-57.3] | < 0.001 |
| Base ECV (%) | 29.0[24.5-34.0] | 35.8[29.8-46.0] | 0.029 |
| Mid Native T1 (ms) | 1057[1021-1094] | 1127[1070-1155] | 0.007 |
| Mid T2 (ms) | 44.4[42.7-46.0] | 59.5[53.0-62.8] | < 0.001 |
| Mid ECV (%) | 29.0[24.6-32.5] | 38.8[34.3-43.1] | < 0.001 |

Table S3 Caption: Tissue characterization parameters in T2 ROIs. Continuous variables presented as median [IQR]. Wilcoxon signed-rank test was used to detect a difference between groups for continuous variables. ECV, extracellular volume fraction; ROI, region of interest.
